# Interactions of cyclodextrins and their derivatives with toxic organophosphorus compounds

**DOI:** 10.3762/bjoc.12.23

**Published:** 2016-02-05

**Authors:** Sophie Letort, Sébastien Balieu, William Erb, Géraldine Gouhier, François Estour

**Affiliations:** 1 Normandie Université, COBRA, UMR 6014 et FR 3038, Université de Rouen, INSA de Rouen, CNRS, 1 rue Tesnière, 76821 Mont-Saint-Aignan Cedex, France

**Keywords:** cyclodextrin, enzyme mimic, nerve agents, organophosphorus compounds, pesticides

## Abstract

The aim of this review is to provide an update on the current use of cyclodextrins against organophosphorus compound intoxications. Organophosphorus pesticides and nerve agents play a determinant role in the inhibition of cholinesterases. The cyclic structure of cyclodextrins and their toroidal shape are perfectly suitable to design new chemical scavengers able to trap and hydrolyze the organophosphorus compounds before they reach their biological target.

## Introduction

Due to their biological activities, organophosphorus compounds have had considerable commercial impacts during the last decades, phosphates, phosphonates, phosphinates, or phosphines being prominent in several fields. As an example, drugs bearing an organophosphorus moiety hold a significant place in chemotherapy for the treatment of various diseases. Nevertheless, the high polarity of these molecules may compromise their activity as poor distribution or passive diffusion across cell membranes are the usual drawbacks associated with their use. Developed after World War II, organophosphorus compounds were used as antitumor [1–8], antiviral [9–12], antihypertensive [13–14] or in rheumatology [15–17]. Some derivatives of pharmacological interest show an anticholinesterase activity [18–21], but few of these molecules are used in therapy due to the difficulties to control their inherent toxicity. Nowadays, the main applications of them are to struggle against the pests or in the military. While the administration of organophosphorus drugs is carefully controlled, improper use of pesticides and chemical warfare agents, the so called “nerve agents”, can cause fatal poisonings through cholinesterase inhibition in tissues and brain [20].

**Figure 1:**
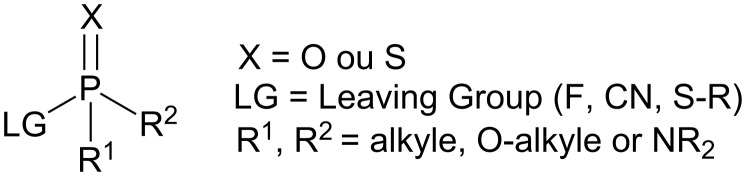
Structure of NOPs.

Neurotoxic organophosphorus (NOPs) share a typical structure with a pentavalent phosphorus atom linked to a leaving group (LG), two substituents (R^1^ and R^2^) and an oxygen or a sulfur atom (Figure 1), explaining the wide structural diversity of organophosphates.

**Figure 2:**
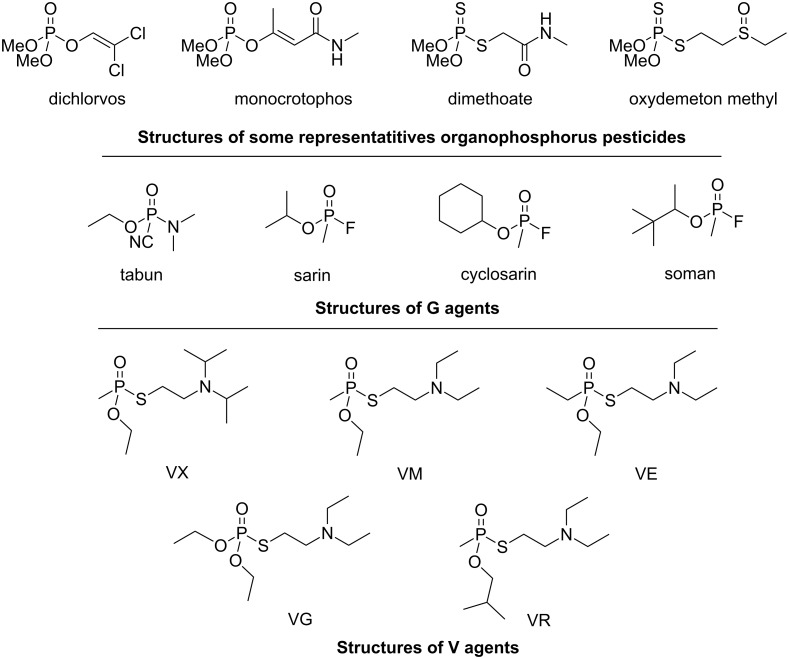
Examples of structures of NOPs.

They can be classified into three categories depending on the nature of the leaving group. The first one includes the least toxic compounds used as pesticides; the second one corresponds to the G agents developed as chemical warfare agents and the third one to the dreadful V agents group (Figure 2).

Due to their use in agriculture, organophosphate accidental or voluntary intoxications are common [21–26]. Moreover, despite the chemical weapons convention, the use of chemical warfare agents in asymmetric wars or in terrorist attacks remains a permanent threat. A key challenge for military forces and rescue services is to have efficient tools to detect and to degrade organophosphate nerve agents [27]. In this respect, cyclodextrins (CDs) are interesting scaffolds because of their macrocyclic structure able to bind more or less selectively some organic substrates via non covalent interactions [28–30]. Due to their affinity for organophosphorus compounds, CDs have gained a significant attention for a variety of applications such as sensors for detection [31–32] or materials for nerve agents decontamination [33].

Various analytical methods exist to monitor the NOPs exposure [34–36]. However, these are long, expensive, required trained staff and are therefore unsuitable for field implementation. An appealing approach to overcome these drawbacks relies on the use of CD-based chemosensors. Apart from detection, the main concern in any event of exposure remains the fast degradation of NOPs into less toxic metabolites by rapid hydrolysis. For this purpose, a key strategy is to design chemical scavengers based on CDs, acting as enzyme mimics. Contrary to bioscavengers, the use of synthetic compounds allows an access to various derivatives and avoids the highly expensive biotechnological methods, the stability issues and the potential immunogenic risks associated to proteins such as hydrolases [37].

This review focuses on the various aspects of the detoxification process mediated by CD derivatives: (1) the ability of CDs and their derivatives to form inclusion complexes with organophosphorus pesticides and G nerve agents; (2) the hydrolysis of the nerve agents by CDs under specific conditions; (3) the selective modifications of these cyclic oligosaccharides to improve their hydrolytic efficiency at physiological pH and temperature.

## Review

### Inclusion studies between CDs and pesticides

**Figure 3:**
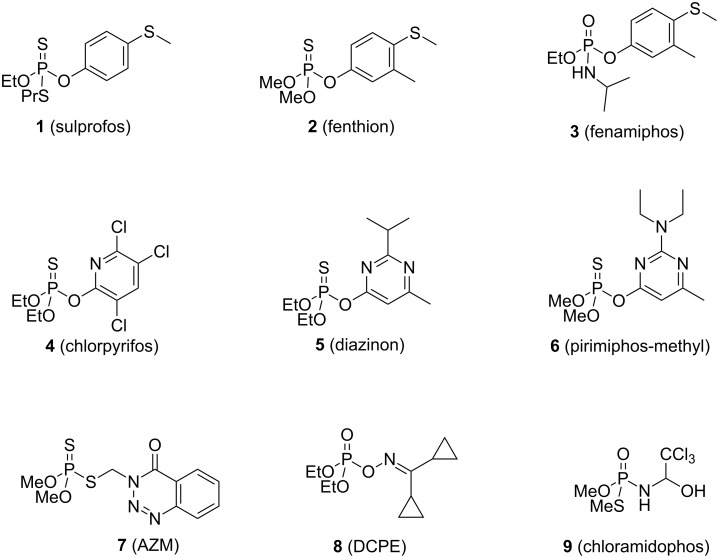
Structures of pesticides studied in the literature as guest to form an inclusion complex with CDs.

Due to their toroidal structure, CDs are able to interact with organophosphorus compounds. It is well known that many synthetic pesticides (compounds **1**–**9**, Figure 3) can form inclusion complexes with CDs, often resulting in modifications of their chemical and physical properties [38–39]. Thus, valuable information on the stoichiometry, the stability and the mode of entry of the guest into the host could be obtained by the characterization of the inclusion complexes in solution or in solid state. These three parameters have to be considered because they strongly determine the potential of CDs as scaffolds for the development of enzyme mimics. Various analytical tests were used to access such information in the cases of pesticides [40–42]. Phase solubility diagrams could be obtained if changes of their water solubility upon inclusion occur, whereas differential scanning calorimetry (DSC) could be used to discriminate inclusion complexes from external associative complexes. UV-visible and fluorescence spectroscopy often provided useful information, while IR was rarely used. Finally, NMR and X-ray crystal structures gave more details on the inclusion mode and the interactions of the pesticide into the oligosaccharide cavity. In addition to these characterization methods, molecular modelling analysis can afford further clues to select the suitable CD derivative for one specific pesticide. This could also support the rational design of new macrocyclic compounds able to strongly interact with organophosphates. The structure of the substrate plays a predominant role in the inclusion phenomenon. In this review, pesticides **1**–**9** were classified according to their more or less close similarities.

#### Pesticides bearing a methyl(phenyl)sulfane group

The interactions between β-CD and sulprofos (**1**, Figure 3) were investigated using various technics [43]. Despite the pronounced solubility changes consistent with complex formation, little variations in the UV spectra, combined with the guest’s small chemical shifts observed in ^1^H NMR experiments (0.01–0.06 ppm), suggest the formation of external complexes rather than an inclusion complex. However the inclusion complex formation was strengthen by molecular modeling studies with the aromatic group included into the cavity. Finally, DSC curves showed a first endothermic portion starting at 225 °C, typical of a melting process, and a second sharp exothermic decomposition peak, proving the formation of the inclusion complex. Thus, on the basis of these last results, the formation of an internal inclusion complex was favored by the authors.

In 2013, Cruickshank reported the X-ray crystal structures of the heptakis(2,3,6-trimethyl)-β-cyclodextrin (TRIMEB)–fenthion complex (complex with **2**, Figure 3), as well as isothermal and non-isothermal thermogravimetric studies [44]. The complex crystallizes in the space group *P*2_1_2_1_2_1_, with the dimethyl phosphorothioate unit located close to the primary rims of the TRIMEB cavity, in its usual tetrahedral conformation. The alkylsulfanyl group is localized out of the larger rim of the TRIMEB cavity, the aromatic group slightly twisted out of the rim. In addition to the structural elucidation of this complex, isothermal and non-isothermal thermogravimetric analysis were performed. Obtained from the solid-state kinetic experiments, the relatively high energy barrier for complex dissociation revealed the thermal stability conferred upon guest encapsulation within the CD molecules.

NMR experiments allow the enantiomers discrimination in presence of a chiral additive to create visible diastereomeric species. ^31^P NMR spectroscopy in presence of CDs as chiral selectors has therefore been employed to discriminate the enantiomers of fenamiphos (**3**, Figure 3) in quantitative water analysis [45]. Neutral CDs such as β-CD, methyl-β-CD, hydroxyethyl-β-CD (HE-β-CD), hydroxypropyl-β-CD (HP-β-CD), and hydroxyethyl-γ-CD (HP-γ-CD) were evaluated in these experiments. β-CD and HP-β-CD were selected as the best chiral solvating agents with an approximately equal level of enantiomeric discrimination. This occurs through the intermolecular inclusion of the fenamiphos phenyl ring within the apolar CD cavity, as confirmed by 2D nuclear Overhauser spectroscopy (ROESY) analysis.

#### Pesticides bearing a nitrogen heterocycle

The association constants of chlorpyrifos (**4**, Figure 3) with β-CD, HP-β-CD, and 6-*O*-α-maltosyl-β-CD hydrate (G2-β*-*CD) in aqueous media were determined by photochemically induced fluorescence (PIF, Table 1) [39,46]. Results are summarized in Table 1. A 1:1 stoichiometry with close complexing ability was found for β-CD and HP-β-CD [46], while a 1:2 complex was found to be predominant in the case of G2-β*-*CD [39]. These results can be corroborated with the aqueous solubility studies of chlorpyrifos in the presence of α-CD, β-CD, γ-CD, HP-β-CD and G2-β*-*CD [39] as a solubility increase is only observed with G2-β*-*CD. The respective association constants values calculated from the phase solubility diagram were 12 M^−1^ for *K*_1_ (1:1 complex) and 3895 M^−1^ for *K*_2_ (1:2 complex), in good agreement to those obtained by PIF, suggesting an effective sequential binding of two CD molecules to one chlorpyriphos molecule.

**Table 1:** Association constants of chlorpyriphos determined by PIF [39,46].

	*K*_a_ (M^−1^)		

	β-CD	G2-β-CD	HP-β-CD

*K*_1_^a^	90 ± 28	12.34 ± 3.1	116 ± 19
*K*_2_^b^		3895 ± 183	

^a^*K*_1_: association constant for a 1:1 complex. ^b^*K*_2_: association constant for a 1:2 complex.

Compared to other pesticides, the low *K*_1_ values observed for chlorpyrifos with α-CD and β-CD may lie in the steric hindrance resulting from the three chlorine atoms, which may inhibit the complexation. No correlation was found between the binding constant values and the hydrophobicity of the pesticides, suggesting that steric effects are probably predominantly involved in complex formation.

The inclusion of the apolar heterocyclic ring into the CD cavity is the most likely situation to occur. γ-CD has the largest secondary alcohols rim radius whereas the β- and γ-CDs’ primary alcohols cones are similar. The guest molecule’s inclusion depth corresponds to the distance from the phosphorus atom to the secondary OH rim. As this distance is the highest with γ-CD, diazinon (**5**, Figure 3) is more deeply inserted [47]. According to these results, the high association constant observed with γ-CD is mainly due to a good fitting of the pyrimidine ring into the cavity, while one ethoxy substituent protruding from the lower rim. In the cases of α- and β-CD, the phosphoryl residue is located outside the cavity. For α- and β-CD, the methyl substituent is more or less deeply inserted into the cavity, pointing downwards, while it is located largely on the secondary rim of γ-CD, pointing upwards. Finally, the isopropyl group is mainly outside, directed upwards for α- and β-CD, whereas located on the secondary rim of γ-CD.

The effect of β- and HP-β-CD aqueous solutions upon the fluorescence properties of pirimiphos-methyl (**6**, Figure 3) were also investigated [46]. As the inclusion phenomena occur, it is reported that the fluorescence properties of the guest are improved compared to its free form in aqueous solution. For both β- or HP-β-CD, the increase of their concentrations resulted in a progressive blue-shift and a fluorescence increase of pirimiphos-methyl. A 1:1 stoichiometry model was found, and the fitting of the observed fluorescence intensity versus the initial CD concentration by a non-linear regression analysis allows the authors to estimate the binding constants of 171 M^−1^ and 736 M^−1^ for β- and HP-β-CD, respectively.

In presence of HP-β-CD, the fluorescence properties of azinphos-methyl (AZM, **7**, Figure 3) were improved by a factor of three but even if it induces a higher fluorescence enhancement than β-CD, this remains too weak to use this technique for routine analysis [48]. Finally the binding constant of the host-guest inclusion complex was reported at 690 ± 140 M^−1^.

#### Pesticides bearing a non-aromatic group

X-ray powder diffraction analysis and DSC validated the formation of a complex between (diethoxyphosphinoximino)dicyclopropylmethane (DCPE, **8**, Figure 3) and γ-CD [49].

Complexes of DCPE with α-, β-, γ- and HP-β-CD were also analyzed [50], and their stoichiometry coefficients evaluated. The NMR spectrum showed an upfield shifts of the internal CD’s hydrogens (H-3 and H-5), whereas chemical shifts of H-1, H-2, and H-4 protons remained identical in presence of β-, γ- and HP-β-CD. For the α-CD complex, none of the protons of the CD showed a significant shift, probably due to a shallow complex formation with the DCPE remaining on the rim of the α-CD cavity. The authors suggested that one ethoxy group could enter into the CD cavities with a stoichiometry coefficient of 1:1 for β-, γ- and HP-β-CD, and 1:2 for the DCPE:α-CD complex.

The stability of these complexes were kinetically analyzed through the DCPE degradation monitoring in aqueous solution in the presence or absence of CD derivatives [49–50]. The degradation rate remained unaffected for α- and β-CD complexes whereas a decelerating effect for γ- and HP-β-CD was noticed, suggesting a pronounced stabilizing effect of these two CDs on DCPE. γ- and HP-β-CD could then be able to include the DCPE molecule or its reactive group deeper within their cavity, shielding it from hydrolysis, than α- and β-CD could.

β-CD:chloramidophos (**9**, Figure 3) complex was formed by a kneading method [51] and UV spectrophotometry analysis revealed a 1:1 stoichiometry and a binding constant of 203.0 M^–1^ for this complex. FTIR analysis revealed the disappearance of the absorbance band at 804 cm^−1^, due to a possible intermolecular inclusion of the -CCl_3_ group into the β-CD. Additionally, C–N stretching vibration band shifted from 1426.6 cm^−1^ to 1425.2 cm^−1^, as a result of hydrogen bonding between the nitrogen atom of the guest and hydroxy functions of the host. Additionally, the presence of a guest might weaken the hydration water binding, as revealed in DSC experiments by the lower endothermic fusion peak, specific to a dehydration process. X-ray powder diffraction analysis finally confirmed the complex formation.

All these studies proved that organophosphorus pesticides are able to interact with CDs and their derivatives. The affinity between the host and the guest strongly depends on the oligosaccharidic structure, and any modification of the CDs scaffold could lead to significant changes of the inclusion complexes’ stability. The structure of the pesticide also plays a major role in the mode of inclusion. For instance, aromatic ring as substituent of the phosphorus atom are often included into the cavity, while steric hindrance is able to impair the complex formation. Finally, without aromatic groups, the mode of entry of the pesticide depends on the specific affinity of the phosphorus substituents for the CD cavity.

### Transformations of NOPs mediated by CDs

According to the above mentioned results, CDs provide interesting scaffolds able to bind organophosphorus compounds. The complex stabilization between the substrate and the oligosaccharide relies on weak interactions such as hydrophobic interactions, hydrogen bonding and van der Waals forces. In some cases, the formation of a stable inclusion complex results the guest’s hydrolysis reaction rate increase.

#### Degradation/hydrolysis of pesticides

**Figure 4:**
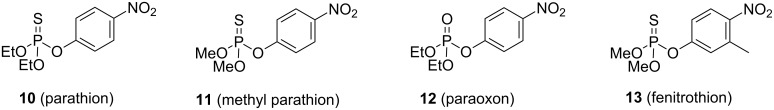
Structures of pesticides sensitive to the presence of CDs.

The inclusion of organophosphorus pesticides into the CDs cavity could either promote or prevent their hydrolysis and photodegradation. Thus, many studies were performed to rationalise the outcome of the reaction between CDs and organophosphorus pesticides (compounds **10**–**13**, Figure 4).

**Scheme 1:**
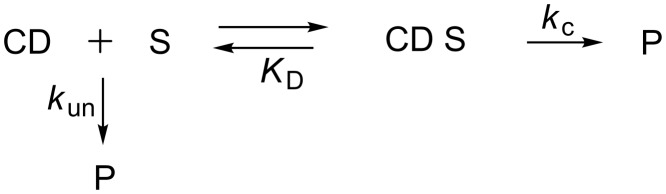
The hydrolysis mechanism of substrate (S) in presence of a cyclodextrin (CD).

The influence of CDs on the hydrolysis rate of parathion (**10**, Figure 4), methyl parathion (**11**, Figure 4) and paraoxon (**12**, Figure 4) was evaluated [52]. In buffer solution (pH 8.5) with humic acids, the authors first noticed a saturation effect on the hydrolysis rate when the CD concentration was increased, suggesting the formation of a CD-pesticide inclusion complex (CD S) prior to the pesticide hydrolysis (Scheme 1).

However, the outcome of this complexation strongly depends of the host–guest system. Indeed, α-CD displays a weak inhibitive effect on parathion hydrolysis whereas a mild promotive effect is noticed for methyl parathion and paraoxon hydrolysis. β-CD inhibits parathion and methyl parathion hydrolysis but promotes paraoxon ones whereas an inhibitive effect of the three hydrolysis was noticed in presence of γ-CD (*k*_c_/*k*_un_, Table 2).

It was previously established that the catalytic effect of CDs strongly depended on the interactions between the included pesticide and the secondary hydroxy groups of CDs. The correlation between inclusion parameters and catalytic properties of CDs was studied by induced circular dichroism (ICD) of the inclusion complexes using rotational-strength analysis method. ICD analysis underlines a change of the environment in the cavity due to the formation of the intermolecular complex. Positive and negative bands observed are respectively attributed to the axial and equatorial positioning of the organophosphorus compound. For the three hosts, the *para*-nitrophenoxy group is included with its long-axis parallel to the cavity. The study shows that the ratio *k*_c_/*k*_un_ has not an accurate correlation with the dissociation constant of the inclusion complex (*K*_d_, Table 2) but it seems that the different inclusion depths of the pesticides into the CD cavity could explain these variable results (α_in_, Table 2).

**Table 2:** Kinetic and equilibrium parameters for CD-organophosphate pesticides systems (25 °C, pH 8.5, phosphate buffer containing 0.1% (w/v) humic acids) [52].^a^

	Substrates	*t*_1/2,c_ (day)	*t*_1/2,un_ (day)	*k*_c_/*k*_un_	*K*_d_ × 10^3^ (M)	α_in_ × 10^10^ (cgs units)

α-CD	parathion	16	13	0.78	1.7	0.45
	methyl parathion	8.5	9.6	1.13	5.2	0.30
	paraoxon	4.2	5.2	1.23	12.0	0.62

β-CD	parathion	90	13	0.14	3.5	2.90
	methyl parathion	88	9.6	0.11	1.9	2.48
	paraoxon	3.7	5.2	1.39	8.4	1.01

γ-CD	parathion	21	13	0.61	8.1	2.12
	methyl parathion	18	9.6	0.53	5.5	1.95
	paraoxon	5.8	5.2	0.89	9.2	1.54

^a^*t*_1/2,c_ and *t*_1/2,un_: half-life times of the included and free pesticides; *k*_c_ and *k*_un_: first-order rate constants for the hydrolysis of the included and free pesticides respectively (Scheme 1); *K*_d_: dissociation constant of the inclusion complexes; α_in_: inclusion-depth parameter estimated by induced circular dichroism with a pesticide concentration of 5.2 × 10^−5^ M and a CD concentration of 8.5 × 10^−3^ M; α_in_ is expressed in cgs (centimeter–gram–second) units.

It was reported that the catalytic activity is affected by the hydrogen bonding between the secondary OH groups of the CD and the reactive site of the guest. Indeed, such interactions increase the nucleophilicity of the secondary hydroxy groups and facilitate their attack onto the phosphorus atom. Indeed, when the *para*-nitrophenoxy moiety is deeply included into the cavity (parathions/β-CD, parathions/α-CD and paraoxon/γ-CD systems), the phosphorus atom is too far from the secondary hydroxy groups, is also shielded from the hydroxide ions and the pesticide’s hydrolysis is thus inhibited. On the contrary, a moderate inclusion of the *para*-nitrophenoxy moiety into the CD cavity (paraoxon/β-CD system) implies a sufficient proximity between the phosphorus atom and the secondary hydroxy groups, thus promoting the pesticide hydrolysis. In the case of pesticides/α-CD systems, the relatively weak inhibition (parathion/α-CD) or catalysis effect (methyl parathion/α-CD and paraoxon/α-CD) imply a shallow inclusion of the phosphorus atom. It might be generalized that the inhibitive or promotive effect of the native CDs strongly depends on the inclusion depth of the guest, which can modify its interaction with the catalytically active secondary hydroxy groups or the hydroxide ions in the solvent.

Acting as the active site, the substitution of secondary hydroxy groups might impact the hydrolysis rate. This was investigated comparing the effect of β-CD, DIMEB and TRIMEB on the alkaline hydrolysis of parathion, methyl parathion, and fenitrothion [53–54]. In all host–guest systems, the rate of alkaline hydrolysis is reduced with the the inclusion of the organophosphorothioate pesticides into the CDs (*k*_c_/*k*_un_ < 1, Table 3).

However it appears clearly that the three studied CDs do not share the same pesticide hydrolysis inhibition level as in all cases, fenitrothion is hydrolyzed faster than parathion and methyl parathion. Indeed the two latter can form deeply included complexes (high α_in_ parameter) compared to fenitrothion which is therefore less shielded from hydroxide ions, leading to the highest hydrolysis rate observed in this series (Table 3) [42]. The origin of this trend was disclosed by theoretical semi-empirical calculations of parathion-, methyl parathion- and fenitrothion-β-CD complexes [55]. It was shown that the methyl group, adjacent to the nitro function slightly impairs both deep and axial inclusion of fenitrothion into the cavity. The methylation degree of the hydroxy groups also influences the catalytic/shielding effect of the CDs in a pesticides dependant manner. For both parathion substrates, the hydrolysis rate of β-CD inclusion complexes is decreased by permethylation, but increased by 2,6-dimethylation. On the other hand, a progressive decrease of the hydrolysis rate of fenitrothion is observed with methylation of β-CD. These unpredictable results revealed that much more complex parameters than the easiness of the guest entry into the (methylated)CD are involved in the hydrolysis. Indeed, the methylation degree could also affect the steric hindrance of the secondary rim, resulting in CD geometrical changes with modifications of the complex stability, the orientation and inclusion depth of the pesticide within the cavity. However, the two main parameters to take into account remain the methylation degree and the pesticide inclusion depth.

**Table 3:** Kinetic and equilibrium parameters for CD-organophosphorothioate substrates systems [53–54].^a^

	Substrates	*k*_c_/*k*_un_^b^	*K*_d_ (10^3^ M)^b^	*k*_c_/*k*_un_^c^	*K*_d_ (10^3^ M)^c^	α_in_ × 10^10^ (cgs units)^c^

β-CD	parathion	0.209	1.9	0.139	3.5	2.92
	parathion methyl	0.186	1.6	0.110	1.9	2.55
	fenitrothion	0.539	2.4	0.660	4.4	0.44

DIMEB	parathion	0.237	3.2	0.274	4.2	1.34
	parathion methyl	0.223	2.0	0.147	2.5	1.89
	fenitrothion	0.518	5.3	0.453	9.8	0.62

TRIMEB	parathion	0.142	3.8	0.104	5.6	0.93
	parathion methyl	0.126	2.7	0.093	4.1	1.26
	fenitrothion	0.355	5.0	0.280	7.2	0.27

^a^*k*_c_ and *k*_un_: first-order rate constants for the hydrolysis of the included and free pesticides (Scheme 1); *K*_d_: dissociation constant of the inclusion complexes; α_in_: inclusion-depth parameter estimated by induced circular dichroism with a pesticide concentration of 1.8 × 10^−4^ M and a CD concentration of 2.1 × 10^−2^ M. α_in_ is expressed in cgs (centimeter–gram–second) units; ^b^parameters of the alkaline hydrolysis measured at 25 °C in NaOH 0.1 M; ^c^parameters of the alkaline hydrolysis measured at 25 °C in phosphate buffer (pH 8.5) containing 0.1% (v/v) humic acids.

This inhibition effect of native β- and γ-CD, DIMEB and TRIMEB on the fenitrothion hydrolysis was also reported by Rougier et al. [56]. The reaction was first carried out without CD at various NaOH concentrations and then with CDs at constant NaOH concentration in water containing 2% dioxane. The kinetic data reveal that β-CD, γ-CD or TRIMEB all inhibit the fenitrothion hydrolysis (Table 4).

**Table 4:** Association constants (*K*_a_) and ratio of the observed rate constants of the fenitrothion hydrolysis.^a^

	*K*_a_ (M^−1^)	*k*^CD^_obs_/*k*_obs_^b^	Ref.

β-CD	417 ± 18	0.68	[56]
γ-CD	99 ± 36	0.70	[56]
TRIMEB	511 ± 31	0.27	[56]
DIMEB	1690 ± 900	0.16	[57]

^a^*k*^CD^_obs_ and *k*_obs_: observed rate constants of fenitrothion hydrolysis respectively in the presence and absence of CD; ^b^values determined in aqueous solution containing 2% dioxane in NaOH 0.5 M at 25 °C with a CD concentration of 0.01 M.

Despite the differences in the association constants for β- and γ-CD, they share a similar level of inhibition (decrease of 30% and 32% of the hydrolysis rate respectively), the best result being obtained with TRIMEB and its 73% reduction of hydrolysis. Another study was later carried out with DIMEB in the same experimental conditions [57] and showed that this CD, characterized by a significantly high association constant, was the strongest inhibitor of fenitrothion hydrolysis (decrease of 84% – Table 4). It clearly appears that methylated β-CDs have a higher hydrolysis inhibition effect than the native ones. Indeed, the steric hindrance resulting from the methyl groups tends to extend the CDs’ cavity size, which allows a deeper inclusion of fenitrothion. Furthermore, the methyl groups also protect the phosphate moiety against external nucleophiles attack by impairing the entry of hydroxide ions.

X-ray analysis of fenitrothion-TRIMEB and -DIMEB complexes showed that the phosphorothioate groups are close to the primary alcohols of the CDs. As a result of intramolecular hydrogen bonds O-2···HO-3’, the DIMEB’s cavity is larger and more prone to form inclusion complexes. The pesticide is thus included deeply into the cavity, protected from nucleophilic attack, leading to a stronger hydrolysis inhibition with DIMEB than with TRIMEB [57]. Contradiction between these conclusions and Kamiya’s results [53–54] were explained by the different reaction conditions and the DIMEB sample employed.

Most of the studies about the pesticides hydrolysis were carried out in alkaline aqueous solutions. However, in the natural environment, the medium pH is usually nearly neutral and the promoting or protecting effects of CDs on hydrolysis might be pH-dependant. Thus, in 1999, Ishiwata and Kamiya reported the effect of α-, β- and γ-CDs on the hydrolysis rate of eight organophosphorus pesticides in neutral aqueous solutions [58]. In sharp contrast to the inhibition of pesticides hydrolysis in alkaline media reported above, at neutral pH, the three native CDs display a catalytic effect on the hydrolysis of all the studied pesticides, highlighting the importance of pH [53–54]. As some hydroxy groups of CDs might be ionized in alkaline media, the authors proposed that the resulting electrostatic repulsions could expand the size of the cavities, resulting in a better hydrolysis inhibition of the pesticides through its deeper inclusion.

The formation of inclusion complexes can also modulate the organophosphorus photodegradation rates, but the results strongly depend on the host–guest systems [59–60] (Table 5).

**Table 5:** Inclusion effect of CDs on the initial photolysis of parathion and paraoxon [59–60].^a^

	pesticides	*k*_c_/*k*_f_^b^	α_in_ × 10^10^ (cgs units)^c^	*k*_c_/*k*_f _^c^	α_in_ × 10^10^ (cgs units)^c^

α-CD	parathion	1.08	0.38	1.13	0.43
	paraoxon	1.12	0.56	1.18	0.58

DIMEA	parathion	/	/	1.02	0.22
	paraoxon	/	/	1.04	0.35

β-CD	parathion	0.46	2.74	0.26	2.97
	paraoxon	1.41	0.92	1.31	0.94

DIMEB	parathion	/	/	0.44	2.18
	paraoxon	/	/	1.31	0.75

γ-CD	parathion	0.69	2.26	0.75	1.86
	paraoxon	0.88	1.43	0.82	1.46

^a^*k*_c_ and *k*_f_: initial photodecay rate of the residual percentage of respectively the included and the free pesticides; α_in_: inclusion-depth parameter estimated by induced circular dichroism (pesticide concentration of 3.9 × 10^−5^ M and CD concentration of 4.8 × 10^−3^ M), ^b^(pesticide concentration of 5.2 × 10^−5^ M and CD concentration of 7.1 × 10^−3^ M); 25 °C in water; ^c^α_in_ is expressed in cgs (centimeter–gram–second) units; 25 °C in water/methanol (80/20, v/v).

The α- and γ-CD respectively exhibit a slight promotive and inhibitive effect on the photolysis of both parathion and paraoxon, whereas a full reverse of reactivity is noticed with β-CD (inhibiting parathion photolysis but promoting paraoxon’s one). Following the same trend as for pesticide hydrolysis, the photolysis outcome is related to the inclusion depth of the pesticide within the CD cavity. For the paraoxon/β-CD complex, the inclusion depth remains moderate, which places the reactive phosphorus center close to the CD’s hydroxy groups. On the contrary, for the parathion/β-CD, paraoxon/γ-CD and parathion/γ-CD complexes, a deep inclusion of the phosphorus atom occurs, preventing its interaction with CD’s hydroxy groups or with water. The comparison between native α- and β-CDs and their methylated analogues reveals that the methylation of hydroxy groups decreases both the inhibitory or promotive effect of CDs, probably due to a higher steric hindrance around the torus.

Later, the effect of native cyclodextrins on the photodegradation of organophosphorus pesticides was evaluated in water containing humic acids [61]. In these conditions, the three native CDs promote the photodegradation of the included pesticides, following the order α < γ < β. It seems that this promotive effect relies on the CDs’ ability to complex both the organophosphorus pesticides and the reactive radical resulting from the photosensitization of humic acids. As the promotion effect of CDs follows the order α < γ < β, it appears that the size of the cavity is not the only parameter to take into account. Indeed, the ability of CDs to relay radicals towards the phosphorus atom might be tuned by the geometrical disposition of the hydroxy groups of CDs. The origin of the higher efficiency of β-CD on the photodegradation might be due to the presence of a strong hydrogen bond network between the secondary hydroxy groups, which restricts their degree of liberty, and lets them to relay the active radicals toward the phosphorus atom.

#### Hydrolysis of chemical warfare agents

Accelerating nerve agent’s hydrolysis, CDs are attractive compounds to achieve their efficient detoxification under physiological conditions. A common feature of these agents is the chiral phosphorus atom and as CDs and their derivatives are also chiral, a stereoselective interaction with the nerve agents is possible. The stereochemistry of organophosphates is to be considered important insofar as the individual stereoisomers of a neurotoxic agent do not share the same level of toxicity [20].

**Figure 5:**
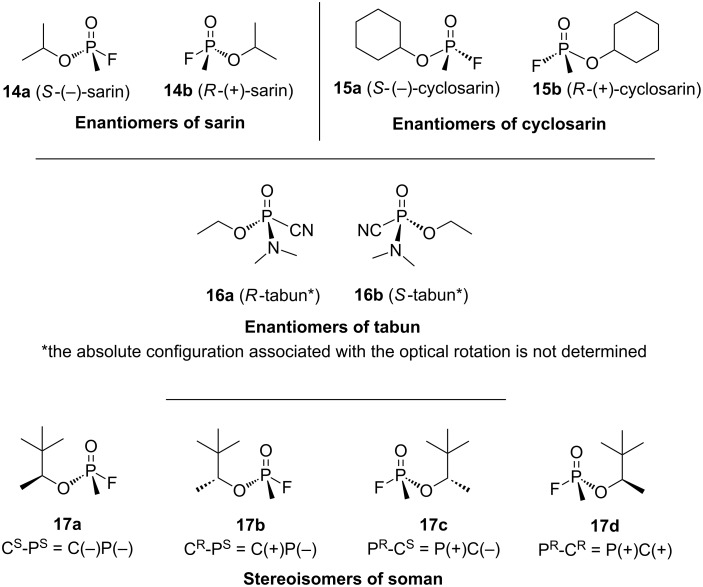
Structures of the different stereoisomers of G agents.

Sarin (compounds **14a** and **14b**, Figure 5), cyclosarin (compounds **15a** and **15b**, Figure 5) and tabun (compounds **16a** and **16b**, Figure 5) consist of a mixture of two enantiomers. Due to the presence of two chiral centers (the phosphorus atom and the carbon atom of the 1,2,2-trimethylpropyloxy group), soman presents four stereoisomers: C(*S*)P(*S*), C(*R*)P(*S*), C(*S*)P(*R*) and C(*R*)P(*R*) (compounds **17a**–**d**, Figure 5).

**Scheme 2:**
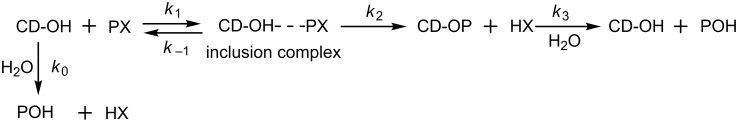
Reaction mechanism of CD accelerated decomposition of organophosphorus compound (PX).

CDs can tune a reaction mechanism in two ways: (a) by creating a covalent bond between the macrocycle and the reagent, first forming an intermediate and then the product; (b) by providing the reactants a new environment with novel reactivity, i.e., selectivity and/or rate due to the apolar CD cavity. In alkaline medium hydrolysis of organophosphorus species, an intermediate mechanism is usually involved. The formation of an inclusion-complex first occurs and then the secondary alkoxide of the host pseudo-intramolecularly attacks the phosphorus atom to form a covalent bond. Finally an alkoxide ion could react with the formed phosphonates to regenerate the CD secondary alkoxide and to release the hydrolyzed product (Scheme 2).

In 1970, van Hooidonk and Breebaart first studied the alkaline hydrolysis of the enantiomers of isopropyl methylphosphonofluoridate (sarin) with α-CD in aqueous solution at pH 9.0 and 25 °C. A hydrolysis rate enhancement is observed and they report the formation of an inclusion complex between the α-CD and sarin prior to the nucleophilic attack of an ionized hydroxy group of the α-CD which becomes phosphonylated [62]. No rate acceleration is noticed with α-methylglucoside, which indicates that the cyclic nature of α-CD and its ability to form inclusion complexes is essential to get a catalytic activity. Stereospecificity, both in the formation of the inclusion complex and in the nucleophilic reaction, are also observed to some extent. According to the authors, the (*S*)-(+)-sarin (**14b**, Figure 5) forms a more stable inclusion complex than the (*R*)-(−)-sarin (**14a**, Figure 5), the latter being more easily hydrolyzed (Table 6). Thus, a better affinity with the CD cavity does not invariably imply a higher reaction rate.

**Table 6:** Dissociation constant (*K*_d_) and rate constant (*k*_2_) for the nucleophilic reaction of the enantiomers of sarin in the presence of α-CD [62].^a^

	*K*_d_ × 10^2^ (M)	*k*_2_ × 10^2^ (min^−1^)

(*S*)-(+)-sarin	0.6 ± 0.04	8.7 ± 0.2
(*R*)-(−)-sarin	4.0 ± 0.6	310 ± 30

^a^Conditions: aqueous solution 0.1 M KCl, pH 9, 25 °C.

The comparison of the dissociation (*K*_d_) and rate constants (*k*_2_) of the two sarin enantiomers clearly shows that the stereospecificity mainly occurs in the nucleophilic attack rather than in the complex formation. Nevertheless, the optical rotations attributed to each enantiomer of sarin are in contradiction to those described by Benschop and De Jong in 1988 [20]. Indeed, they determined that the (*R*)-sarin is dextrorotatory whereas the (*S*)-sarin is levorotatory. Chirality has been attributed following the reviewed rules of Cahn, Ingold and Prelog [63–64] which classified the oxygen atom of the P=O bond as the minor priority compared to the alkoxy substituents [65]. In their 1988 work, Benschop and De Jong stated that the nerve agent stereoisomers’ absolute configuration of the phosphorus atom was not firmly established. Thus, the van Hooidonk and Breebaart’s work dating from 1970, it is highly probable that they did not attributed the correct absolute configurations to the enantiomers of sarin, which means that the (*S*)-(−)-enantiomer would be hydrolyzed faster in presence of α-CD.

They also studied the alkaline hydrolysis of the two enantiomers of isopropyl *para*-nitrophenyl methylphosphonate [65] and of isopropyl (*S*)-2-dimethylaminoethyl methylphosphonothioate [67] in the presence of α-CD at 25 °C. The result indicates that the inclusion complexes stereospecific formation is not significant, but that α-CD is predominantly phosphonylated by the (−)-enantiomers. The stereospecificity observed with sarin, isopropyl *para*-nitrophenyl methylphosphonate and isopropyl (*S*)-2-dimethylaminoethyl methylphosphonothioate is ascribed to better interactions between the included (*−*)-enantiomer and the secondary hydroxy groups responsible of the nucleophilic attack.

In the 80s, Désiré and Saint-André studied the effect of the three native cyclodextrins on the hydrolysis reaction of tabun, VX, sarin and soman [68–70]. It appears that α-, β- and γ-CDs are unable to catalyze the hydrolysis of VX as the rates remain similar in the presence of these CDs compared to the spontaneous hydrolysis rates, even at pH 9 or 11. In contrast, the three native CDs catalyze the hydrolysis of sarin and soman (Table 7). Nevertheless, at pH 7.40, native CDs-catalyzed hydrolysis of sarin is rather slow and only soman seems to be a good substrate, especially for β-CD. The formation of a stable inclusion complex between CDs and the organophosphorus nerve agent is postulated as the first step in the catalytic process.

**Table 7:** Inactivation rate constants (min^−1^) of tabun, sarin and soman in the absence^a^ and in the presence^b^ of CDs [70].^c^

CD concentration		pH	tabun	sarin	soman

/		7.40		(5.4 ± 0.3) × 10^−4^	(5.3 ± 0.3) × 10^−4^
/		9.00	(7.0 ± 0.4) x 10^-3^	(1.4 ± 0.2) × 10^−2^	(8.2 ± 0.2) × 10^−3^

α-CD	10 mM	7.40			(2.2 ± 0.1) × 10^−2^
	20 mM	7.40		(2.5 ± 0.2) × 10^−3^	(2.8 ± 0.2) × 10^−2^
	50 mM	9.00	(7.6 ± 0.8) × 10^−3^		

β-CD	1 mM	7.40			(3.0 ± 0.1) × 10^−2^
	10 mM	7.40		(2.4 ± 0.3) × 10^−3^	
	10 mM	9.00	(7.5 ± 0.9) × 10^−3^	(6.7 ± 0.3) × 10^−2^	

γ-CD	10 mM	7.40			(4.6 ± 0.3) × 10^−3^
	20 mM	7.40			(6.4 ± 0.2) × 10^−3^

^a^*k*_0_ min^−1^: rate constant observed for the hydrolysis of nerve agent PX in the absence of CD; ^b^(*k*_CD_ − *k*_0_) min^−1^ with *k*_CD_: rate constant observed for the hydrolysis of nerve agent PX in the presence of CD; ^c^conditions: aqueous solution with Tris buffer 10 mM at 25 °C.

Furthermore, it appears that the dissociation constants (*K*_d_) of soman/β-CD and soman/α-CD complexes are significantly lower than those of the sarin/β-CD and sarin/α-CD complexes, which indicates a weak binding between sarin and CDs (Table 8). The only difference between sarin and soman is their isopropyloxy and pinacolyloxy moieties and the binding constants of β-CD/soman and β-CD/pinacolyl alcohol complexes were then investigated. The similar data obtained proved that the pinacolyloxy group was at the origin of the strong complexation of soman into the β-CD cavity.

**Table 8:** Dissociation constants of inclusion complexes of α-, β-, γ-CD with sarin and soman [69].^a^

			*K*_d_ (mM^−1^)		

	concentration range of CD	pH	α-CD	β-CD	γ-CD

sarin	2.5–50 (mM)	8.0	40 ± 10		
	1.5–10 (mM)	9.0		4.9 ± 0.7	

soman	7.5–50 (mM)	7.4	18 ± 5		
	0.2–4.0 (mM)	7.4		0.53 ± 0.05	
	2.5–50 (mM)	8.0			5.5 ± 1.1

^a^Parameters measured in aqueous solution with 10 mM Tris buffer at 25 °C.

They then focused their work on the interaction between soman and β-CD due to its greater affinity for β-CD compared to α- or γ-CDs. According to the study, soman forms an (1:1) inclusion complex with β-CD at pH 7.40. After its complexation into the cavity, soman reacts with a nucleophilic oxyanion formed from a secondary hydroxy group of the CD torus. As a result of this pseudo-intramolecular process, at pH 7.40 and 25 °C, the hydrolysis of soman by the monoanionised β-CD is 2600 times faster compared to a hydroxide ion. Different rates of β-CD-mediated hydrolysis for the four soman stereoisomers were observed. Only the chiral phosphorus center is involved in this stereoselectivity and the two most toxic P(*−*)-diastereomers **17a** and **17b** (Figure 5) of soman are degraded faster (Table 9).

**Table 9:** Percentages of different forms of soman hydrolyzed by 10 mM β-CD after incubation times of 20 and 60 min [70].^a^

forms of soman hydrolyzed	20 min	60 min

racemic soman	44%	62%
P(*−*)-soman	70%	97%
P(*+*)-soman	18%	27%

^a^Conditions: 10 mM of β-CD in aqueous solution with 10 mM Tris buffer, pH 7.40, 25 °C.

In 1995, Cabal investigated the hydrolysis reaction of soman with the three native cyclodextrins and especially the reactivity differences between P(*S*) and P(*R*) isomers at 25 °C and pH 9 [71]. They reported a stereospecific hydrolysis of soman with α-, β- and γ-CDs depending on the absolute configuration of its phosphorus atom. Indeed, more stable inclusion complexes are observed with the P(*S*)-stereoisomers of soman, but the degradation of the P(*R*)-stereoisomers remains strongly faster (Table 10).

**Table 10:** Maximum hydrolysis rate constants *k* and dissociation constants *K* of inclusion complexes of CDs with P(*R*) and P(*S*) stereoisomers of soman [71].^a^

	*k*_R_ (min^−1^)	*k*_S_ × 10^2^ (min^−1^)	*K*_R_ × 10^4^ (M^−1^)	*K*_S_ × 10^4^ (M^−1^)

α-CD	4.87 ± 0.61	3.40 ± 0.10	252.0 ± 0.46.0	17.30 ± 2.10
β-CD	3.07 ± 0.22	3.75 ± 0.14	14.4 ± 2.9	5.35 ± 0.74
γ-CD	0.26 ± 0.01	1.08 ± 0.05	58.6 ± 0.6	6.33 ± 1.80

^a^Conditions: constants measured in 0.01 M NaOH aqueous solution at 25 °C and pH 9.

In agreement with the results discussed above, the stereospecific process is not the inclusion within the α-, β- or γ-CD cavity but the nucleophilic attack which can be rationalized by the different orientations of the P(*R*)- and P(*S*)-stereoisomers within the β-CD cavity. The complexation of the P(*R*)-isomers into the CD cavity is followed by the formation of a hydrogen bond between a C-3 hydroxy and the phosphoryl oxygen atom of soman. Then a C-2 ionized hydroxy function attacks the phosphorus atom to form a covalent bond with the release of the leaving group (fluoride ion). The different orientations of the phosphorus atom substituents for the P(*S*)-isomers prevent the optimal interaction between the ionized hydroxy group and the phosphorus atom and thus reduce the hydrolysis rate.

The hydrolysis rate constants of soman with α- and β-CDs are similar and higher than the one observed with γ-CD which has the largest cavity (Table 10). Thus, the higher dissociation and the lower rate constants for γ-CD indicate that a too large cavity, compared to the guest size, decreases the strength of the inclusion complex. The substrate is therefore not sufficiently enclosed in the cavity to allow the optimal nucleophilic attack by a hydroxy group of CD. The study also reports the influence of the pH on the degradation rate. As the pH is increased, the hydroxy groups of CD are gradually deprotonated, resulting in faster hydrolysis rates for all soman’s stereoisomers.

The hydrolysis of sarin and cyclosarin in presence of β-CD were then studied at 25 °C and pH 9 and also compared with the hydrolysis of soman. Their enantiomers are not degraded at the same rate, the P(*R*)-isomers of sarin and cyclosarin reacting faster than P(*S*)-isomers at pH 9 (Table 11).

**Table 11:** Maximum hydrolysis rate constants *k* and dissociation constants *K* of inclusion complexes of β-CD with methylfluorophosphonates [71].^a^

	*k*_0_ × 10^2^ (min^−1^)	*k*_R_ (min^−1^)	*k*_S_ × 10^2^ (min^−1^)	*K*_R_ × 10^4^ (M^−1^)	*K*_S_ × 10^4^ (M^−1^)

soman	1.46 ± 0.08	3.07 ± 0.22	3.75 ± 0.14	14.4 ± 2.9	5.35 ± 0.74
sarin	2.30 ± 0.11	0.33 ± 0.02	5.92 ± 0.09	9.61 ± 4.9	4.98 ± 0.22
cyclosarin	1.81 ± 0.07	11.00 ± 0.70	5.40 ± 0.30	14.50 ± 0.2	2.52 ± 0.50

^a^Conditions: constants measured in aqueous 0.01 M NaOH solution at 25 °C and pH 9; ^b^*k*_0_: rate constant of the spontaneous hydrolysis.

The different alkoxy substituents on the phosphonate group for the three methylfluorophosphonates do not result in fundamental changes of the dissociation constants. However, the rather slow hydrolysis rate of P(*R*)-sarin can be explained by the space arrangement of this stereoisomer into the β-CD cavity. Sarin is the smallest of the three methylfluorophosphonates studied. After the complexation of P(*R*)-sarin, its phosphorus atom is located in the cavity above the level of the catalytically active hydroxy groups of β-CD, whereas for cyclosarin and soman, the phosphorus atoms lies in the plane of the hydroxy groups. The deep inclusion of P(*R*)-sarin into the β-CD cavity could therefore be at the origin of the rather low reaction rate observed for this stereoisomer.

Thus, in all the studies of Cabal, the P(*R*)-stereoisomers of soman, sarin and cyclosarin are faster hydrolyzed than the P(*S*)-stereoisomers in presence of CDs. Nevertheless, due to the contradiction with the hydrolysis rates of soman previously by Désiré and Saint André [68,70], we can again raise a doubt about the validity of the assigned absolute configurations. Indeed, they reported a faster degradation of the two P(*−*)-diastereoisomers whose absolute configuration of the phosphorus atom is assigned to *S* according to Benschop and De Jong’s work [20]. Moreover, Cabal referred to van Hooidonk and Breebaart‘s work [62] to corroborate his results about the hydrolysis rate of sarin. However, we previously underlined the incoherencies between the optical activity and the absolute configurations of the enantiomers of sarin described by van Hooidonk and Breebaart. Consequently, it seems that Cabal inversed all the absolute configuration of soman, sarin and cyclosarin enantiomers, which would imply a faster degradation of the P(*S*)-stereoisomers.

**Scheme 3:**
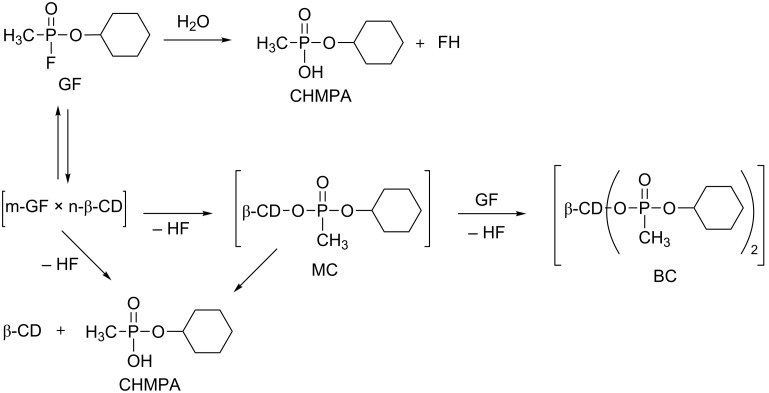
Proposed degradation mechanism of cyclosarin by β-CD [72].

Several studies discussed the interactions between cyclodextrins and organophosphorus compounds and their influence on the hydrolysis rates, but the reaction pathways are not always clearly understood. In 2013, Kranawetvogl et al. studied the degradation of cyclosarin (GF) by β-CD to propose a mechanism [72]. CHMPA (*O*-cyclohexyl methylphoshonic acid), which is the product of the GF hydrolysis but also stable mono and binary covalent CHMP-β-CD conjugates (MC and BC). Thus, after the initial inclusion complex formation, the GF can be directly hydrolyzed releasing CHMPA or it can be attacked by a hydroxy group of the CD leading to the MC conjugate. Then, two reaction pathways are possible. The MC conjugate could either be hydrolyzed to regenerate the free unsubstituted β-CD or it could react with another molecule of GF to give the stable binary CHMP-β-CD conjugate (BC) (Scheme 3).

Thus, a competition between a “catalytic pathway” and a “stoichiometric pathway” occurs, the favoured reaction depending on several factors such as the concentrations of β-CD and GF, the concentration ratios of both of them or the use of additional ligands. Indeed, when the concentration ratio GF/β-CD is increased from 1:5 to 1:1, the CHMPA formation decreases. Furthermore, the GF hydrolysis is favoured in Tris-HCl buffer rather than in water. These results clearly show the high complexity of the degradation mechanism of an organophosphorus compound by β-CD.

To efficiently catalyse the NOPs hydrolysis, CDs and their derivatives have to be able to stabilize the reaction intermediate. To this regard, if the formation of a stable inclusion complex is likely to improve the reaction rate, the binding mode of the substrate and CD must match to observe a faster reaction. On the contrary, a non-reactive complex would decrease the hydrolysis rate. The design of more reactive CD derivatives is thus a challenging approach to degrade NOPs more effectively.

#### Design of enzymes mimics based on CDs

The studies based on non-functionalized CDs have disclosed their potential as NOPs scavengers. Nevertheless, improvements remained relatively weak considering the results obtained under physiological conditions (pH 7.4, 37 °C). The design of novel macrostructures displaying a higher hydrolytic efficiency under mild conditions is then a stimulating challenge. The main strategies described rely on the introduction of one or several reactive groups against nerve agents on these cyclic oligosaccharides.

**Figure 6:**
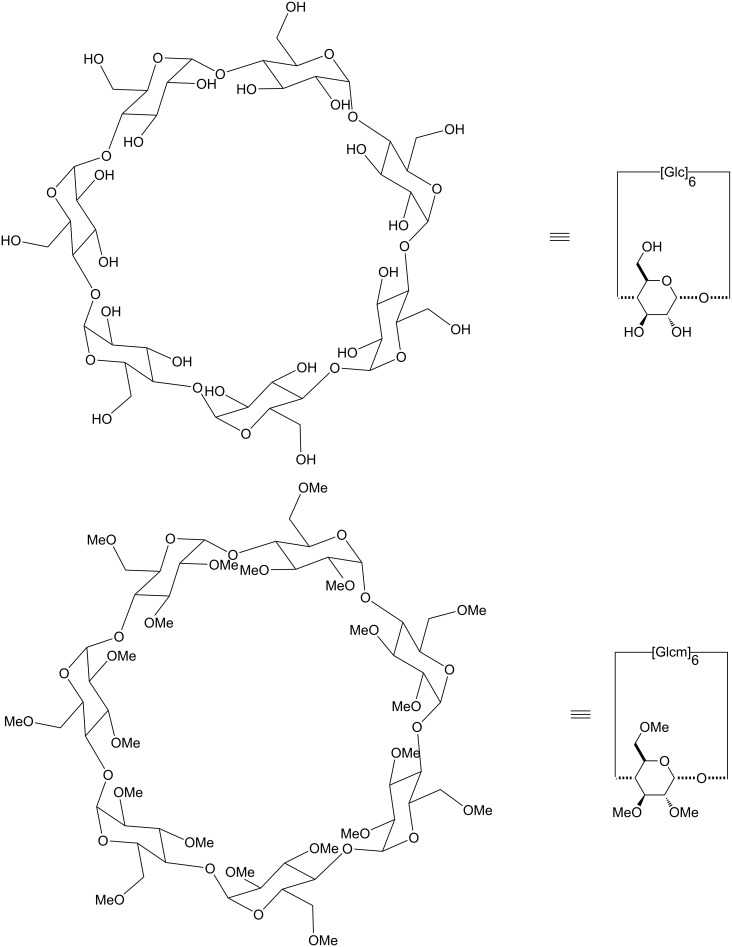
Schematic representations of β-CD and TRIMEB.

As CDs exhibit a large number of primary (C-6 position of glucose units) and secondary (C-2 and C-3 positions) alcohols, selective modifications are therefore difficult. Most of them are based on the slight differences in the reactivity of these three kinds of hydroxy groups. In Schemes 5–10, the CD ring is drawn as in Figure 6 for β-CD and TRIMEB. This representation was also chosen for their derivatives in order to point out the modified units.

#### Modifications at the C-6 positions

**Scheme 4:**
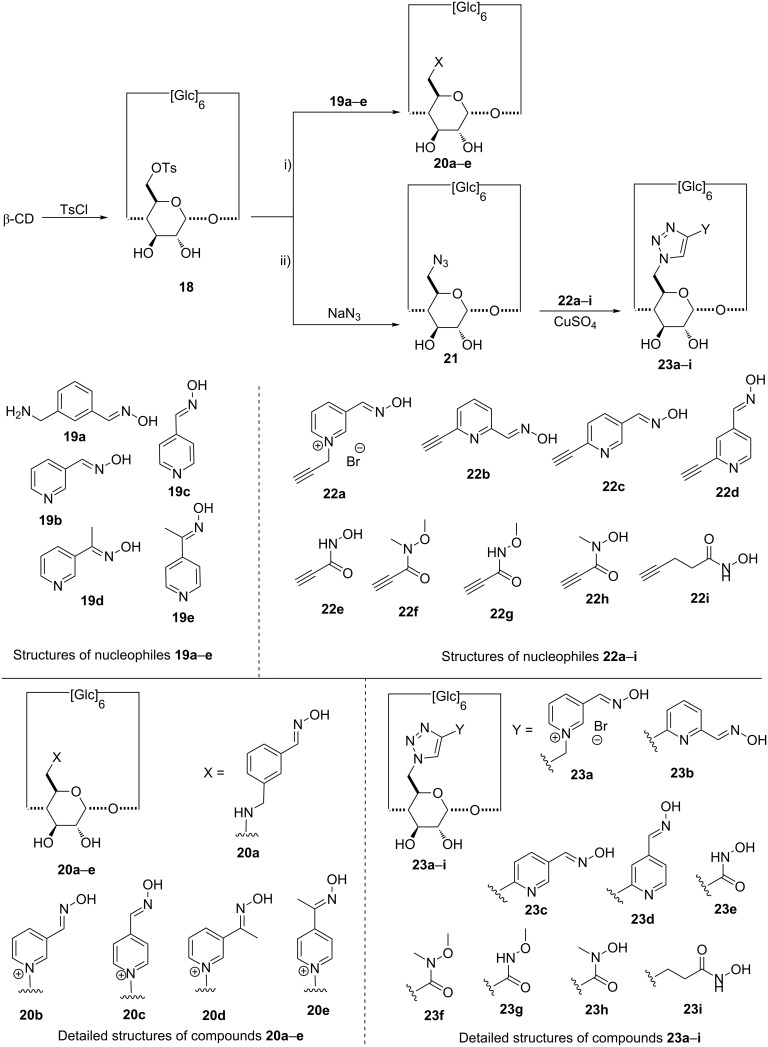
Synthetic pathways to 6-monosubstituted CD derivatives.

**Synthesis:** In 1989, Breslow and Anslyn [73] described the synthesis of a bis(imidazole)-β-CD derivative able to catalyze a 4-*tert*-butylcatechol cyclic phosphate hydrolysis 120 times faster than the simple spontaneous hydrolysis. This seminal work proved unequivocally that modified CDs are able to accelerate the hydrolysis rate [74]. Regarding these preliminary results based on the C-6 position functionalization, Kubik et al. [75] described two routes to access β-CD derivatives monosubstituted on the primary rim with various nucleophiles. The first one is based on the monotosylation of one C-6 hydroxy, followed by a nucleophilic substitution with a substrate bearing an aldoxime group (pathway i, Scheme 4); the second route starts from the same tosylated intermediate **18**, substituted by an azide function, precursor of a triazole ring used to link the aldoxime moiety (pathway ii, Scheme 4). Following this second approach, Kubik et al. published new α-, β- and γ-CD derivatives functionalized with hydroxamic acids on one C-6 position [76].

**Efficiency of compounds 20a–e against chemical warfare agents:** The authors demonstrated the high activity of the pyridinium derivatives, especially the compounds **20b** and **20d**, against cyclosarin (Scheme 4). Indeed, the nerve agent was totally hydrolyzed in less than five minutes in presence of **20b**, the most toxic enantiomer, (−)-cyclosarin **14a**, reacting faster in this enantioselective degradation [75].

Structure–activity relationships based on this first series of compounds revealed the determinant role of oximes’ nucleophilic strength in the hydrolysis process. Firstly, the less active compound is the weak acid oxime derivative **20a**. Then, the formation of the more nucleophilic aldoximate is favored with the pyridinium derivatives **20b**–**d**, explaining their high activity. The moderate efficiency of ketoximes **20d** and **20e**, compared to that of the corresponding aldoximes **20b** and **20c**, strengthen this hypothesis. Finally, the *para*-substituted pyridinium derivative **20c** is much less reactive than the *meta* compound **20b**. This tends to prove that even slight structural differences have a major impact on the hydrolytic reactivity of the cyclodextrin.

Based on these results, Kubik et al. then evaluated the hydrolytic activity of **20b** on various alkyl methylphosphonofluoridates [77]. As **20b** was more active than native β-CD for sarin derivatives hydrolysis, they were able to clearly establish the role of the nucleophilic group. Contrary to sarin derivatives, soman detoxification by **20b** was not as effective, suggesting a non-reactive position of the nucleophilic oxime to efficiently react with this toxic.

Worek et al. also demonstrated the prophylaxis effect associated to **20b** in the case of anesthetized guinea pigs poisoned with cyclosarin [78]. A dose of cyclosarin s.c. (100 μg/kg, ≈2LD_50_) led to rapid death, except for the animals treated with an injection of **20b** i.v. (100 mg/kg) 5 min prior to cyclosarin s.c. For them, the systemic signs of poisoning almost completely disappeared. Reiter et al. investigated the detoxification mechanism of **20b** and demonstrated a stereoselective hydrolysis of the most toxic (−)-cyclosarin [79]. As previously described, the formation of an intermediate inclusion complex is likely to occurred. The affinity between cyclosarin and **20b** could be explained by (1) ion–dipole interaction between the pyridinium group of **20b** and the phosphoryl oxygen of cyclosarin and (2) the cyclohexyl moiety inclusion into the hydrophobic CD cavity. The formation of such an inclusion complex could be followed by the fast hydrolysis of cyclosarin or the formation of mono-, bis-, tris- and tetrakis-conjugates which may ultimately be decomposed into small organic fragments. The scavenger **20b** is therefore consumed during the cyclosarin degradation and thus could not be considered as a catalyst.

If **20b** was the most efficient compound for the cyclosarin detoxification of this pyridinium series, Kubik et al. showed that **20c** was more efficient for the degradation of tabun (Scheme 4) [80]. However, it should be pointed out that a simple glucose derivative bearing the same nucleophilic group has a similar activity. The efficiency of **20c** is therefore clearly related to the pyridinium oxime functional group and not to the inclusion properties of the macromolecule.

**Efficiency of compounds 23a–i against chemical warfare agents:** Amongst the derivatives **23a**–**i** with the pyridine aldoxime linked to the CD by a triazole ring, compound **23a** is the most interesting scavenger. To access more potent compounds, Kubik et al. later introduced two α-nucleophiles on A and D units of β-CD [80]. Surprisingly, only a slight increase of the activity against tabun compared to that of **20b** was noticed. The steric hindrance induced by the two aldoxime groups might explain this disappointing result. It is also possible that the two reactive groups are not in optimal positions due to the rigidity of the triazole-based spacer.

Introduction of hydroxamic acids via a Huisgen reaction on α-, β- and γ-CD also leads to scavengers sharing the same level of efficiency than a glucose unit bearing the same functionnal group. These surprising results proved that for these derivatives, the macrocycle has no influence on the degradation of the nerve agent. This might be rationalized by the inclusion of the triazole ring into the CD’s cavity by a tumbling phenomenon as shown by Monflier et al. [81]. This process would therefore prevent any interaction of the hydroxamic acid group with the oligosaccharide. *per*-Substitution of the primary face of β-CD improves the activity, but without linear correlation with the number of groups introduced [76].

#### Modifications at the C-2 and C-3 positions

**Scheme 5:**
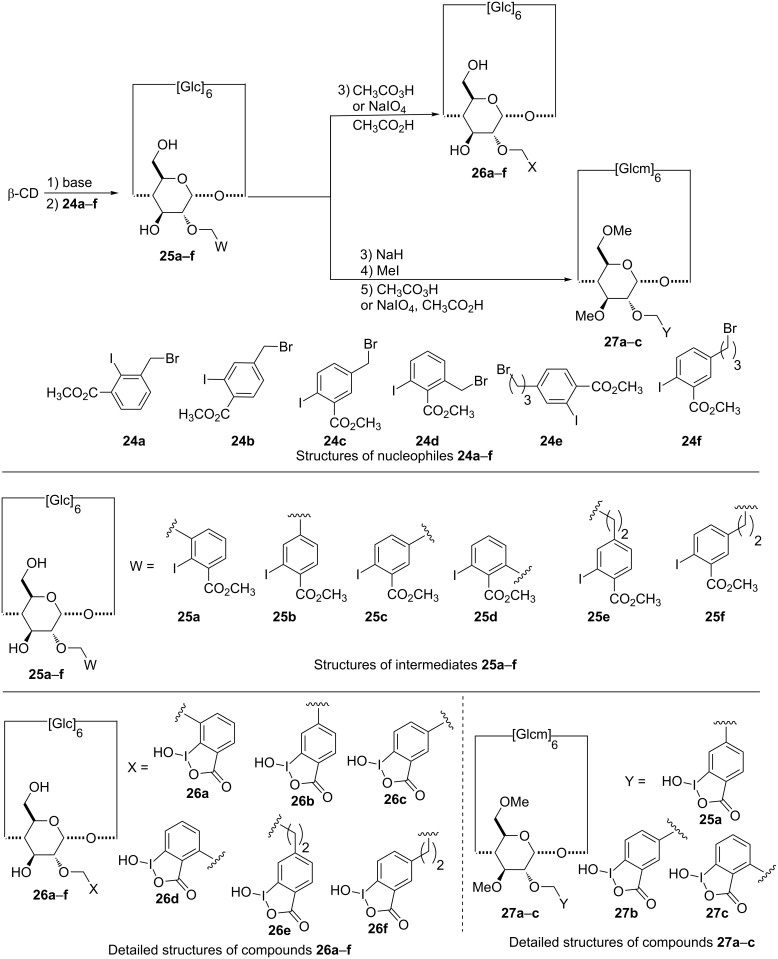
Synthetic pathways to 2-monosubstituted CD by an iodosobenzoate group.

**Synthesis of 2-monosubstituted β-CD derivatives:** A three-step protocol is usually followed to monofunctionalize the most acidic hydroxy groups on the 2-position: (1) deprotonation of that position with a strong base, (2) addition of the required halogenated derivative, (3) activation of the α-nucleophile supported by the CD (Scheme 5). The use of methyl iodobenzoates with variable alkyls linker (one or three carbon atoms) led to a series of CD derivatives supporting a iodosobenzoate group as the α-nucleophile (compounds **26a**–**f**) [82–83]. Careful optimisation studies were carried out to improve the yield of this challenging mono-substitution [83–84]. A wide range of strong bases and their amount (versus equivalents of CD and the halogenated derivative) were evaluated in dimethyl sulfoxide. As expected, halogenobenzylic derivatives **25a**–**d** gave the best yields compared to those obtained with methyl bromopropyliodobenzoates **25e**–**f**. The hydroxy groups of compounds **25b**–**d** were also methylated (compounds **27a**–**c**) to evaluate the influence of the CD hydroxy groups in the organophosphorus substrate degradation [83].

**Scheme 6:**
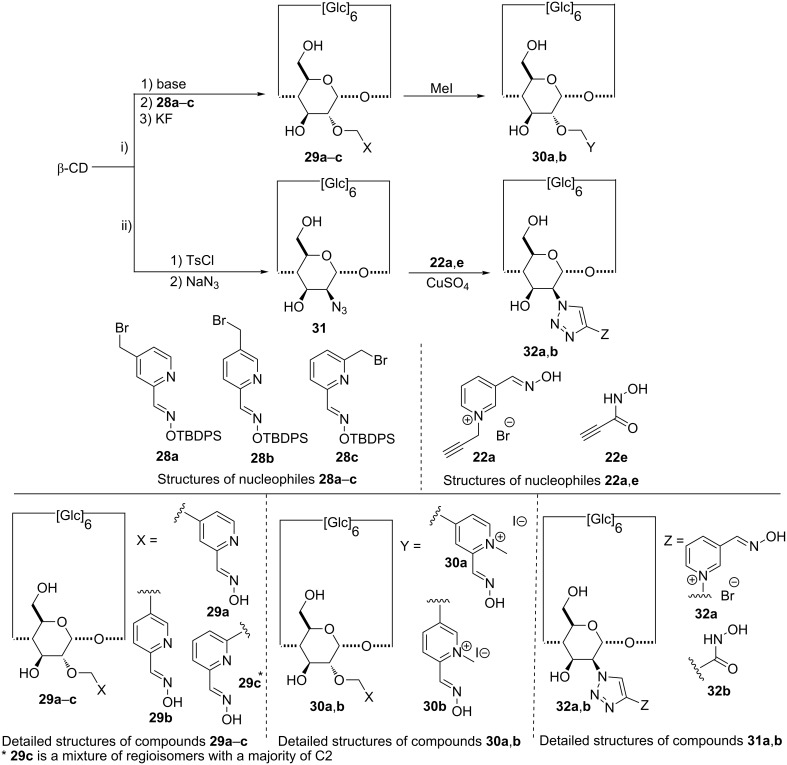
Synthetic pathways to 2-monosubstituted CDs with N–OH derivatives.

It should be mentioned that this selective substitution of β-CD was also found to be substrate-dependant. As an example, the use of pyridinium aldoximates instead of iodosobenzoates (compounds **29a**–**c**, **30a** and **30b**, Scheme 6), required modified conditions.

The use of non-conventional activation methodologies (i.e., ultra-sound or microwaves) proved to be valuable to access some of these challenging CD derivatives [85]. Introduction of an oxime group at the C-2 position was also performed with a cooper(I)-catalyzed azide–alkyne cycloaddition between the 2’-azido-β-CD **31** and the alkyne derivatives **22a**,**b** to access the corresponding monosubstituted CD **32a**,**b** [76].

**Scheme 7:**
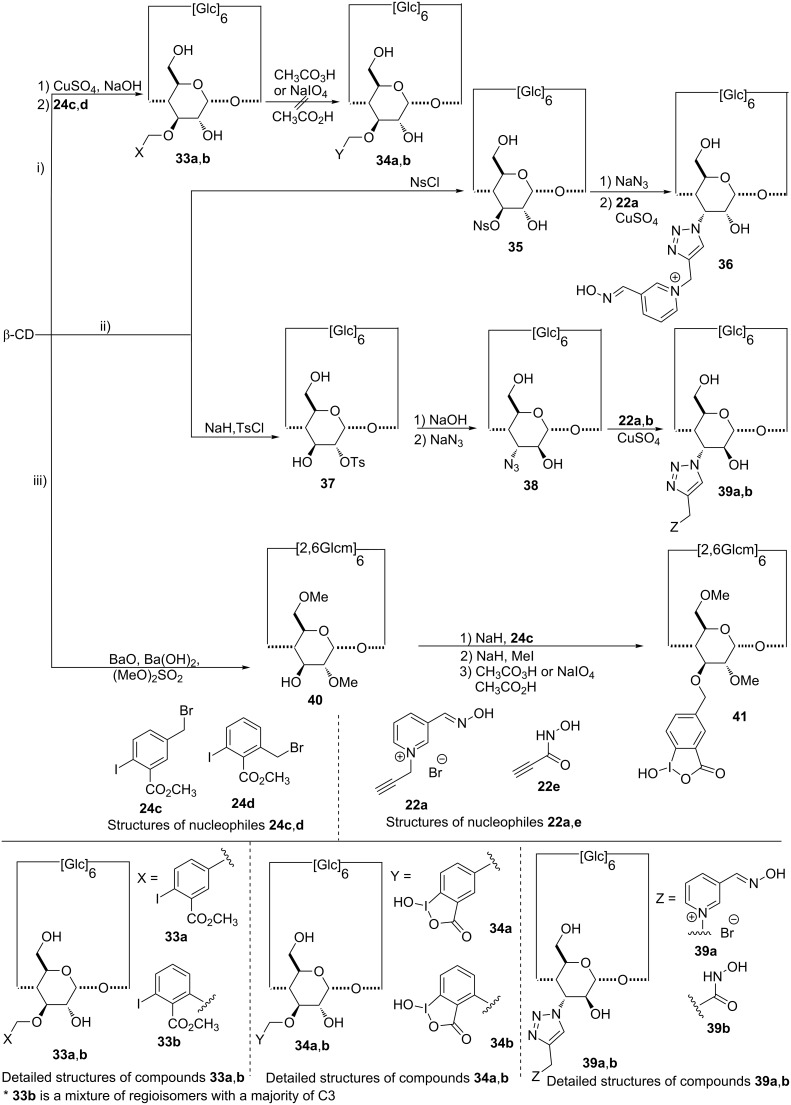
Synthetic pathways to 3-monosubstituted CDs.

**Synthesis of 3-monosubstituted β-CD derivatives:** As the facial position of the reactive groups might modify the catalytic efficiency of the CD derivative, the monofunctionalization of the C-3 alcohols was carried out [86]. The temporary complexation of specific β-CD secondary hydroxy functions allows the monofunctionalization of the C-3 position through the formation of a copper(II)–β-CD complex. A diagonal link between copper ions and C-2 and C-3 oxygen atoms of adjacent glucopyranose units might distort the CD cavity, making the hydroxy groups on position 3 more accessible. Moreover, as this complex is formed in aqueous sodium hydroxide, the hydroxy groups could be deprotonated in situ to perform the subsequent substitution reaction in a one-pot manner. Methyl iodobenzoates were introduced following this approach (Scheme 7, **33a**,**b**), unfortunately, degradation occurred during the final oxidation step to obtain the α-nucleophile group [87]. The 3-monosubstituted derivative **41** was successfully obtained starting from the partially-protected 2,6-dimethyl-β-CD (Scheme 7) [88]. Finally, CDs bearing an oxime (**36** and **39a**) or a hydroxamic acid (**39b**) group in position 3 were prepared as previously described via an azide–alkyne cycloaddition (Scheme 7) [76,80].

**Scheme 8:**
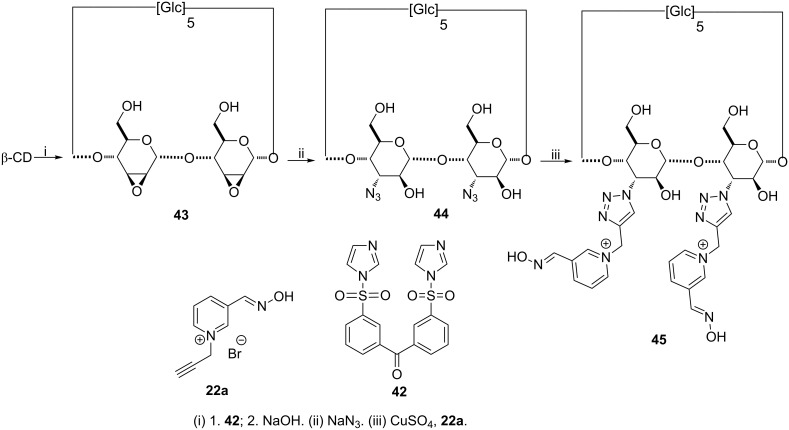
Synthetic pathways to 3-homodisubstituted CDs.

**Synthesis of difunctionalized β-CD derivatives:** As the hydrolytic activity on NOPs may differ depending on the number and the nature of the groups introduced on CDs, methodologies were developed to regioselectively access disubstituted β-CDs. An elegant strategy to homodifunctionalize the A and B units’ secondary hydroxy group of the β-CD rely on the use of the tethered disulfonylimidazole **42** to access a key intermediate easily converted to the corresponding di-2,3-mannoepoxido compound **43** (Scheme 8) [89]. The 3^A^,3^B^-diazido-3^A^,3^B^-dideoxy-bis(*altro*)-β-cyclodextrin **44** was then obtained by reaction of **43** with sodium azide and a copper(I)-catalyzed azide–alkyne cycloaddition finally afforded the desired disubstituted compound **45** [80]. This compound is not stricto sensu a CD derivative as already the case for 2- and 3-monosubstituted CDs **32a**,**b**, **36** and **39a**,**b**.

**Scheme 9:**
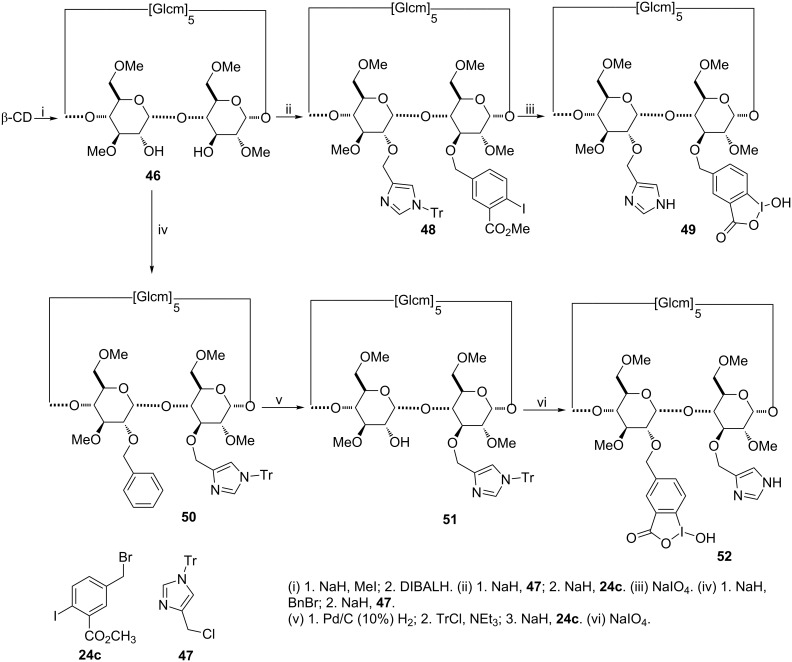
Synthetic pathways to 2,3-heterodisubstituted CDs.

Another particularly convenient strategy to introduce two different groups on adjacent units of methylated β-CD relies on the use of DIBAL-H to produce 2^A^,3^B^-diol **46** from TRIMEB (Scheme 9) [90]. The two remaining free alcohols were then functionalized with the α-nucleophile and an imidazole group [88]. The acid–base dyad thus obtained is likely to improve the hydrolysis of nerve agents. The imidazole group was first introduced by a regioselective substitution at C-2, followed by a C-3 benzylation to access compound **49**. As the other regioisomer **52** could not be obtained so easily, additional protection–deprotection steps were required.

**Efficiency of compounds 26a–f and 27a–c against chemical warfare agents:** Preliminary tests were carried out with paraoxon to evaluate the efficiency of these scavengers as the hydrolysis of this organophosphorus pesticide releases *para*-nitrophenol that is easily monitored by UV–visible spectrophotometry at 400 nm [82]. It first appeared that the position of the spacer arm on the aromatic ring modifies the efficiency of the scavenger. The greatest hydrolysis rate was observed with the ether oxide in *para* position to the iodoso function in compound **26c**, while compound **26a** has no effect. According to the paraoxon concentration, a saturation effect of kinetics was observed during the pesticide hydrolysis with the three active compounds **26b**–**d**. The authors proposed that the *para*-nitrophenolate remains included into the cavity, thus poisoning the CD derivatives **26b**–**d**.

Compound **26c** was therefore selected to evaluate its efficiency against chemical warfare agents using a biological enzymatic assay [91]. The ability and the detoxification rate decreases in the order cyclosarin > sarin > tabun and no activity is observed against VX. The detoxification process of tabun is performed in only one step when sarin and cyclosarin required two. The first one led to a fast reduction of the acetylcholinesterase (AchE) inhibition, followed in a second phase by a slow decrease until complete recovery of AchE activity. Two different phenomena could explain this behavior. The first one might be the formation of an inclusion complex between the NOP and the CD, each toxic characterized by different levels of affinity as guest in the torus. Thus VX and tabun would not form any inclusion complex whereas sarin and cyclosarin will, preventing the AChE inhibition. As a result, cyclosarin is almost fully trapped by the scavenger whereas 70% of the enzyme benefits of this phenomenon with sarin. In 2011, Reiter et al. confirmed the fast high-affinity binding of cyclosarin on **26c** as well as synergistic effects able to markedly accelerate the inactivation rate of cyclosarin. Moreover, they provided kinetic constants and correlation coefficients of the inactivation of cyclosarin after incubation with **26c** [92]. The second phase postulated above would finally rely on the hydrolytic activity of the iodosobenzoic acid group.

It should be noted that tabun, soman and cyclosarin have different CD-mediated reduction rates of their AchE inhibitory potential [83,93]. The acetylcholinesterase inhibitory potential of cyclosarin and soman significantly depends on the link position on the aromatic group, while tabun remained less affected.

The reduction of the AChE inhibitory potential of cyclosarin and soman is faster with the propylene-linked derivatives **26e** and **26f** than with the methylene-linked permethylated counterparts **27a** and **27b**, while their efficiencies were similar against tabun [82]. This result for tabun is in agreement with those obtained with compounds **26a**–**d**, showing no significant interaction between the nerve agent and the CD cavity. It also appeared that compounds **26c** and **26f** share a similar efficiency against cyclosarin whereas a weaker activity was observed for **27b**. If the α-nucleophile group undoubtedly plays a major role in the hydrolysis of cyclosarin, the free hydroxy functions of the CD also have an influence, whereas the distance between the iodosobenzoate substituent and the macrocycle could be considered as a minor parameter. Regarding the stereoselectivity issue, compound **26e** showed a marked preference for the (−)-cyclosarin **14a** degradation, while no stereoselective GF detoxification was recorded with derivative **27f** [93].

**Efficiency of compounds 29a–c, 30a,b, 32a,b, 36 and 39a,b against chemical warfare agents:** In the case of compounds **29a**–**c**, the degradation of cyclosarin was always found to depend on the position of the linker on the aromatic ring [85]. Fast degradation of the nerve agents was achieved for compound **29c** with a 2,6-disubstituted pyridine ring. No general trend in quaternarization emerges as **30a** was more active than **29a**. Moderate enantioselective hydrolysis of cyclosarin in favour of the levorotary isomer was obtained with compounds **29b** and **29c**. Interestingly, all compounds of this series are less efficient than the derivative **26c** bearing an iodosobenzoate substituent.

Every compound, **32a**, **36** and **39a**, including respectively a mannose, an allose or an altrose substituted unit, are able to hydrolyze tabun faster than derivative **23a**, bearing the same aldoxime group in position 6 [80]. The most efficient scavengers are then obtained with the α-nucleophile group on the secondary face. On the contrary, moving the hydroxamic acid to the secondary face of the CD reduces their efficiency against tabun (compounds **32a** and **32b** versus compound **23e**) [76]. However, in this case, no influence of the CD ring was highlighted for the 6-monosusbtituted CD **23e**, suggesting that the accessibility of the hydroxamic acid group on the secondary face of β-CD is more difficult.

**Efficiency of compounds 45, 49 and 52 against chemical warfare agents:** Introduction of two oximes in position 3 on two adjacent units with compound **45** has almost no effect on tabun hydrolysis compared to its monosubstituted counterpart **39a** [81]. Indeed, the resulting steric hindrance might prevent the cooperation of the second substituent, and/or the triazole-based linker would not allow the required positioning of the two substituents.

The degradation of soman by heterodifunctionalized derivatives **49** and **52** was monitored by ^1^H NMR [88]. If the main product formed is pinacolyl methylphosphonic acid, as already observed in phosphate buffer [94], only compound **49** is able to accelerate the stereoselective hydrolysis of soman. The relative positioning of iodosobenzoate and imidazole groups importance was further highlighted by the lack of activity improvement with the regioisomer **52**. The true protective efficiency of the new scavenger **49** was also proved with an enzymatic assay. Finally, the synergistic effect of these two different groups must be emphasized for their efficiency in both hydrolysing the NOP and protecting AChE, compared to the corresponding 3-monosusbtituted CD **41**. This is the first example of a 2^IB^,3^IA^-heterodifunctionalized β-CD derivative, acting as an enzyme mimic.

## Conclusion

As a result of their toroidal shape, CDs are able to trap organophosphorus pesticides and nerve agents. However, host’s and guest’s structures are both particularly determinant as they can modify the complex stability, the mode of entry and the inclusion depth of the organophosphorus substrate into the macrocyclic cavity. The potential degradation of toxics is then modulated by mixed effects. Further functionalization of CDs could lead to new derivatives with increased NOP hydrolytic potential. Through the introduction of an α-nucleophilic group on the oligosaccharidic torus, nerve agents were significantly hydrolyzed by CD derivatives under physiological conditions. Finally, it was proved that the introduction of two different groups on adjacent units of a permethylated-β-CD resulted in a synergic effect on soman hydrolysis. This new strategy is then a promising way to access original scavengers with an enzymo-mimetic activity.
